# Case report: Reconstruction of the left renal vein with resected autologous right renal vein interposition after excision of an inferior vena cava leiomyosarcoma

**DOI:** 10.3389/fsurg.2022.913927

**Published:** 2022-07-26

**Authors:** Xiaohang Li, Baifeng Li, Na Zhang, Fengshan Wang, Chengshuo Zhang, Ning Sun, Jialin Zhang

**Affiliations:** Department of Hepatobiliary Surgery, First Affiliated Hospital, China Medical University, Shenyang, China

**Keywords:** leiomyosarcoma, inferior vena cava, reconstruction, renal vein, interposition vascular graft

## Abstract

**Background:**

Leiomyosarcoma of the inferior vena cava (IVC) was a rather rare disease with the characteristics of invading the adjacent viscera. Surgical resection is the only potential curative treatment, and radiation therapy and chemotherapy for leiomyosarcoma are not definite. There is few literature reporting the leiomyosarcoma of the IVC.

**Case presentation:**

A previously healthy 64-year-old female was admitted to the First Affiliated Hospital of China Medical University with the complaint of right lower quadrant abdominal pain for almost three years and worsening with a radiating ache in the waist recently. Contrast-enhanced computed tomography(CT) scans revealed a large (7.8 cm*5.5 cm*5.0 cm) irregular hypodense retroperitoneal mass with heterogeneous enhancement and invasion of the IVC, and the right ureter was compressed with proximal ureteral dilatation and hydrops. Three-dimensional CT of the IVC revealed that the IVC was encircled by the tumor with moderate invasion. During the operation, the tumor was resected *en bloc* with the IVC (from the suprarenal to infrarenal segment), the right kidney with ureter, and the duodenum seromuscular layer. As the left renal vein was involved, it was also partly resected. IVC reconstruction was performed with the interposition of a 20 mm diameter polytetrafluoroethylene (PTFE) prosthesis, and the right renal vein was anastomosed between the left renal vein and the reconstructed IVC to guarantee the left renal vein reflux. The patient had an uneventful recovery process with normal renal function after the operation. However, follow-up CT indicated that the left renal vein was blocked two weeks after the surgery. The patient was discharged two weeks after the operation. She continues well and has no evidence of disease fourteen months after the surgery.

**Conclusions:**

Wide excision of the tumor *en bloc* with the IVC is the main treatment for leiomyosarcoma of the IVC. IVC reconstruction with prosthetic PTFE grafts is recommended. When the left renal vein is partly resected due to involvement of the tumor, reconstruction of left renal vein should also be performed to avoid renal impairment. If the right renal vein does not show tumor involvement, the resected right renal vein can be used to reconstruct the left renal vein.

## Background

Leiomyosarcomas are rare sarcomas that arise in the smooth muscle components of the middle layer in veins, usually in the inferior vena cava (IVC). Leiomyosarcoma of the IVC, accounting for 5%–10% of soft tissue sarcomas (1), usually presents with an extremely poor prognosis. The tumor can grow either intraluminally, extraluminally or both with one of the patterns being predominant, although the extraluminal type has been considered the most frequent (2). Since surgical resection is the only potential curative treatment at present, the prognosis depends directly on the extent of the surgical resection. Leiomyosarcoma of the IVC usually invades the adjacent viscera (3); therefore, multiple organ resection and reconstruction of the IVC are usually required for radical excision. Undoubtedly, the complexity of the operation increases the difficulty and complexity of surgical resection and is a major challenge for both vascular and general surgeons. Subsequently, postoperative complications may also increase. Here, we report a case of leiomyosarcoma of the IVC treated with complex surgery and presenting with an uneventful postoperative recovery.

## Case presentation

**Figure 1:**
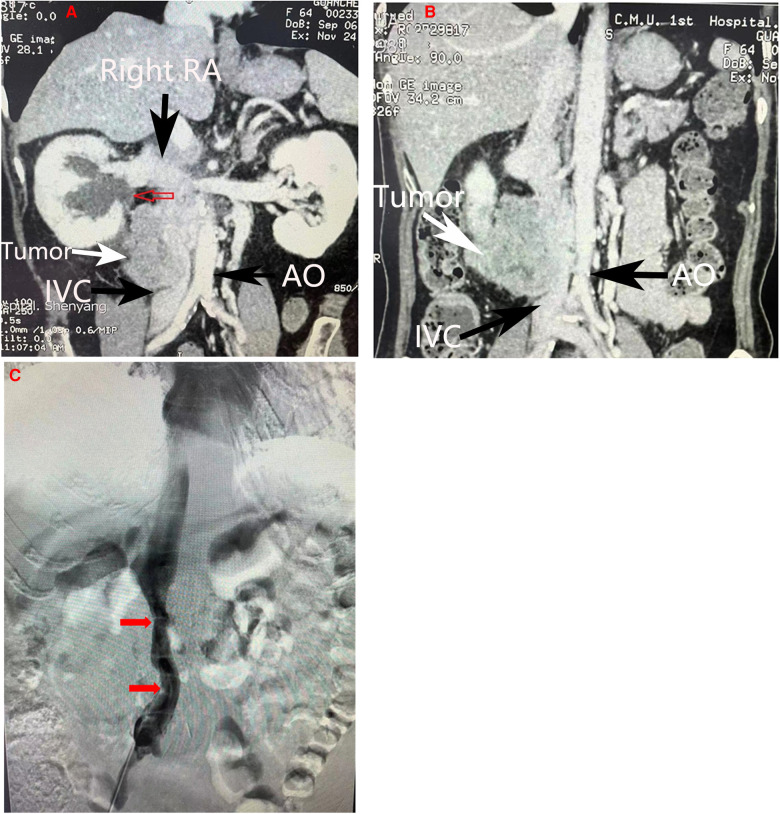
Imaging manifestations. (**A**) Coronal section of abdominal CT scan demonstrating compression of the right ureter with proximal ureteral dilatation and hydrops(red arrow). (**B)** Coronal section of abdominal CT scans howing a heterogeneous mass invading the inferior vena cava. (**C**) Inferior vena cava angiography revealing compression and narrowing of the inferior vena cava(red arrow). The main vessels were marked by arrows in other colors. RA, Right artery; IVC, inferior vena cava; AO, Aorta abdominalis.

A previously healthy 64-year-old female was admitted to the First Affiliated Hospital of China Medical University (Shenyang,Liaoning Province, China) with a large retroperitoneal tumor. The patient had been experiencing right lower quadrant abdominal pain for almost three years, but she had ignored the symptoms until the abdominal pain intensified with a radiating ache in the waist. During physical examination, a large, fixed mass with a diameter of 10 cm could be felt in the right upper abdomen without obvious haphalgesia. The patient presented with normal blood pressure and no swelling in either lower extremity. General laboratory work-up results were within normal limits (Blood routine test: white blood cells, 7,000/L, hemoglobin, 11.1 g/dl; serum creatinine, 0.48 mg/dl; urine routine test: white blood cells: 9.9/µl, red blood cells:19.40/µl; neurone specific enolase: 50.20 ng/ml). The tumor was further diagnosed by following imaging studies, including computed tomography (CT) and endoscopic ultrasonography (EUS), which were performed to investigate the abdominal pain. Contrast-enhanced CT scans revealed a large (7.8 cm * 5.5 cm * 5.0 cm) irregular hypodense retroperitoneal mass with heterogeneous enhancement and invasion of the IVC, and the right ureter was compressed with proximal ureteral dilatation and hydrops (Figure 1A). EUS revealed a hypoechoic mass outside the distal wall of the descending duodenum. EUS-guided biopsy confirmed the diagnosis of a mesenchymal tumor with low malignancy potential. Dimensional CT of the IVC was performed to investigate the relationship between the tumor and the vessel. CT revealed that the IVC was encircled by the tumor with moderate invasion (Figure 1B). To further investigate whether the left and right renal veins had been invaded by the tumor, IVC angiography was suggested, revealing that the IVC was compressed and narrowed (Figure 1C). The right renal vein presented with normal imaging findings and no tumor invasion. Due to the lack of essential tools, left renal vein angiography was not performed successfully. To prevent pulmonary embolism resulting from the abscission of the tumor during the operation, an IVC filter was placed prior to the surgery. According to preoperative imaging, the right kidney needed to be resected due to invasion by the tumor. Preoperative emission tomographic imaging of the kidneys indicated that the left glomerular filtration rate(GFR) was 49.8 ml/min, the right GFR was 34.8 ml/min, and the total GFR was 84.6 ml/min. Given that the retroperitoneal tumor with the IVC and adjacent viscera was resectable *en bloc*, the patient underwent surgical resection.

**Figure 2:**
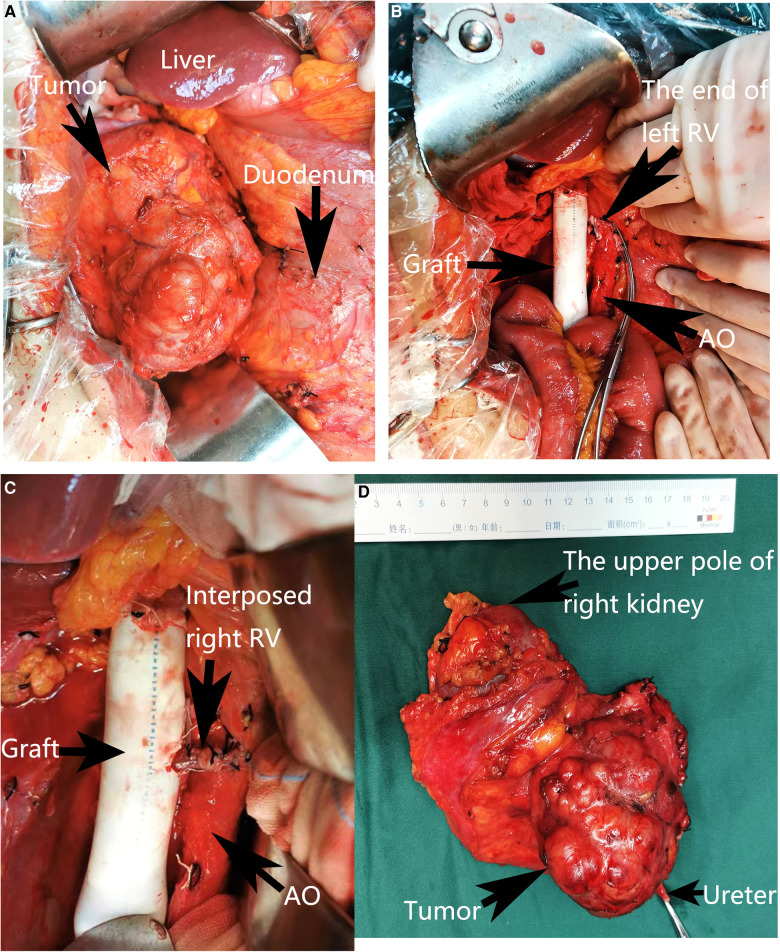
Intra-operative view. (**A**) The view before the excision. (**B**) Reconstruction of the inferior vena cava with an artificial vascular graft. (**C**) Reimplantation of the left renal vein into the artificial vascular graft by interposition of the right renal vein. (**D**) Resection of the tumor carrying the kidney with ureter. The adjacent viscera and main vessels were marked by arrows. RV, Right vein; AO, Aorta abdominalis.

**Figure 3:**
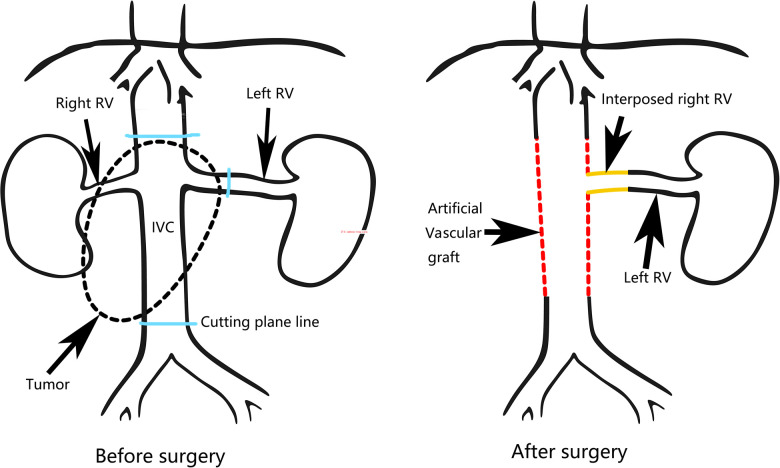
The drawing of the procedure. RV, Right vein; IVC, inferior vena cava.

During the operation, we found that a 6 cm segment of the IVC, the right kidney with ureter, the descending duodenum seromuscular layer and the origin of the left renal vein had been invaded by the tumor (Figure 2A), therefore, the tumor was resected *en bloc* with the IVC (from the suprarenal to infrarenal segment), the right kidney with ureter, and the duodenum seromuscular layer. As the left renal vein was involved, it was ligated and resected to the proximal part of the left renal vein ahead of the genital vein drainage point. IVC reconstruction was performed with the interposition of a 20 mm diameter polytetrafluoroethylene (PTFE) prosthesis (Figure 2B). The proximal and distal IVCs were occluded for 70 min during the operation.There was a high risk of renal insufficiency after surgery for this patient, since a single left kidney without a left vein can not provide sufficient function. After checking the specimen from the right kidney, we found that the right renal vein was not involved. Therefore, we used the resected right vein as a bridge between the reconstructed IVC and the left renal vein. After the right renal vein was trimmed, it was anastomosed between the left renal vein and the reconstructed IVC (Figure 2C). When the vascular occlusion was lifted, left renal vein blood smoothly flowed through the interposed right vein. Figure 2D showed the resection of tumor carrying the kidney with ureter. Since the procedure is a little complicated, Figure 3 showed the drawing of the procedure to visually describe the operation steps. The patient had an uneventful recovery process with normal renal function (serum creatinine 0.71 mg/dl) after the operation, and urine routine test showed normal number of white blood cells (2.5/µl). However, follow-up CT indicated that the left vein was blocked two weeks after the operation, her renal function is still normal(serum creatinine 0.81 mg/dl). Pathological examination verified the diagnosis of leiomyosarcoma. The patient was discharged two weeks after the operation. She continues well with normal renal function (serum creatinine 0.89 mg/dl) and has no evidence of disease eight months after surgery.

## Discussion

Leiomyosarcoma of the IVC was first described by Pearl in 1,871, and fewer than 400 cases have been reported in the literature to date (4, 5).The location of the tumor can divided into three types depending on its relation with the hepatic and renal veins. Type I represents tumors located in the infrarenal IVC, type II represents those in the IVC between the hepatic and renal veins, and type III represents those in the segment above the hepatic veins and up to the right atrium (6). Leiomyosarcoma of the IVC most frequently occurs in the IVC between the hepatic and renal veins. Although leiomyosarcoma of the IVC is a malignant tumor, it usually grows slowly and presents with late clinical manifestations. The symptoms also depend on the location of the tumor. While upper segment tumors may lead to the development of Budd–Chiari syndrome (defined by obstruction of the hepatic outflow tract), tumors located in the middle segment usually lead to right upper quadrant pain, and lower extremity edema occurs more often for tumors located in the lower segment (7). In this case, the tumor was located in the middle and lower segments, and the patient presented with right lower quadrant abdominal pain but no lower extremity edema.

A combination of imaging techniques, such as CT or magnetic resonance imaging (MRI), are useful for the preoperative diagnosis and treatment choice. The literature reports that MRI may achieve greater accuracy in the diagnosis of leiomyosarcoma of the IVC than contrast-enhanced CT (8). In this case, contrast-enhanced CT was first chosen. It clearly demonstrated the tumor's diameter, location and invasion of the surrounding organs. Since it was not clear whether the left and right renal veins had been invaded by the tumor, IVC angiography was further performed. Comprehensive preoperative imaging examinations helped us to determine the tumor's condition and to make plans for guiding the operation. Surgical resection is the preferred treatment. The usefulness of radiation therapy and neoadjuvant or adjuvant chemotherapy for leiomyosarcoma is still controversial (9). Some studies have reported that long-term survival is related to an aggressive surgery (10, 11), as surgical margins are a predictive factor of survival for IVC leiomyosarcoma (12). According to the preoperative imaging and intraoperative exploration, *en bloc* resection of the tumor and the involved IVC(from the suprarenal to infrarenal segments), right kidney and duodenum seromuscular layer with reconstruction of the IVC was performed to obtain tumor-free margins for this patient. Simple IVC ligation may be feasible when collaterals have been established in the presence of long-standing obstruction. Although there were some collaterals in this patient due to preoperative obstruction, simple ligation was not applied. Since the long segments of the IVC (from suprarenal to iliac bifurcation) were affected, the left renal vein would have to be ligated if IVC reconstruction was not performed. The patient’s renal function would be further impaired if left renal vein reflux was blocked after right nephrectomy was performed. Therefore, we performed reconstruction of the IVC with artificial vessels. IVC reconstruction techniques vary from simple suturing/patch repair to synthetic or biological graft interposition based on the extent of caval involvement. If less than 30% of the circumference of the IVC wall is involved, simple suturing in a longitudinal or transverse fashion will usually be feasible (13). A patch repair can be chosen for larger defects. For defects of more than 50% of the vessel, an interposition graft of PTFE or Dacron is usually required (14). Due to the high incidence of thrombosis and stenosis, Dacron is not currently the option of choice (15). In contrast, PTFE grafts provide inert and biocompatible properties and are the preferred option (16, 17). In this case, we used an artificial vessel of PTFE to reconstruct the IVC, and the patient still presented with satisfactory patency without recurrence after a follow-up of 10 months.

IVC reconstruction increases the difficulty and risk of the operation; however, the benefit of IVC reconstruction includes avoiding renal impairment and lower limb edema. Gaignardt et al. considered that ligation of the left renal veins was safe and that the genital veins could provide sufficient collateral drainage and avoid the developmentof renal failure (18). Moreover, no nephrectomy was performed in the abovementioned situation. In our case, right nephrectomy was performed, and drainage of the left kidney should not be impaired. Since the right renal vein did not show tumor involvement, we used it as the interposition graft to extend the left renal vein and perform an end-to-side anastomosis between the interposition graft and the artificial IVC. To our knowledge, no studies have reported this scheme to date. The patient's recovery was very uneventful, with normal renal function and routine urine tests. This verified the key role of the interposition vessel. Although we used low molecular weight heparin (4,100 IU per day) as postoperative anticoagulation, the interposed right renal vein was obstructed by a thrombus, as shown by CT two weeks after the surgery. We considered that the thrombosis was associated with the multiple anastomoses and the long cold ischemia time. The patient’s renal function remained normal after the surgery, and this indicated the patency of the interposed right vein and sufficiency of the renal reflux during the two weeks after the surgery. Renal function was relieved by genital vein drainage after thrombus formation in the interposed right renal vein.

Lopez-Novoa's research showed that renal plasma flow and glomerular filtration rate increased by 34.5% and 17.4% respectively in rats after unilateral nephrectomy. Lee reported that the effecitive average renal plasma flow of the remnant kidney is 317 ml/min in patients after unilateral nephrectomy, and the normal range of effective renal plasma flow was between 210 and 240 ml/min. Therefore, a hyperdynamic circulatory state was confirmed in both rats and human after unilateral nephrectomy. If the remnant renal vein reflux was not sufficient, hypertransfusion may impair the glomerular epithelial cell and result in deterioration of renal function. So it is necessary to guarantee sufficient remnant renal drainage. That is the reason why we interposed autologous right renal vein to restore left renal reflux. It's a pity that the interposition graft did not work two weeks after surgery. However, it bought time for the left kidney to establish collateral circulation. The normal renal function after surgery confirmed the key role of reconstructed left renal vein. If the left vein was not reconstructed, temporary renal dysfunction might happen. It avoided the temporary renal dysfunction. Also, it provided the experience that we should improve the anticoagulation regimen and short the cold ischemia time to decrease the risk of thrombosis in the interposed vessels.

## Conclusion

In summary, wide excision of the tumor *en bloc* with the IVC is the main treatment for leiomyosarcoma of the IVC. Whether the IVC is reconstructed depends on the site of the tumor (especially with renal vein involvement) and whether sufficient collateral venous drainage is established. IVC reconstruction with prosthetic PTFE grafts is recommended, and postoperative systemic anticoagulants after IVC reconstruction are required. If right nephrectomy is performed, venous drainage of the single left kidney should be guaranteed. When the left renal vein is partly resected due to involvement of the tumor, reconstruction of the left renal vein should also be performed to avoid renal impairment. If the right renal vein does not show tumor involvement, the resected right renal vein can be used to reconstruct the left renal vein to maintain sufficient venous drainage.

## Data Availability

The original contributions presented in the study are included in the article/**Supplementary Material**, further inquiries can be directed to the corresponding author/s.
