# Supplementary material for: Case report: Reconstruction of the left renal vein with resected autologous right renal vein interposition after excision of an inferior vena cava leiomyosarcoma
Source: Front Surg. 2022 Jul 26;9:913927. doi: 10.3389/fsurg.2022.913927 (PMC9362845; doi:10.3389/fsurg.2022.913927)
Supplement: Supplementary file 1 [file Data_Sheet_1_v1.pdf]

CARE case report guidelines

# CARE Checklist of information to include when writing a case report

(cc) BY-NC-ND

| Topic | Item | Checklist item description | Reported on Line |
|---|---|---|---|
| Title | 1 | The diagnosis or intervention of primary focus followed by the words "case report" | √ |
| Key Words | 2 | 2 to 5 key words that identify diagnoses or interventions in this case report, including "case report" | √ |
| Abstract (no references) | 3a | Introduction: What is unique about this case and what does it add to the scientific literature? | Abstract, paragraph 1 |
| | 3b | Main symptoms and/or important clinical findings | Abstract, paragraph 2 |
| | 3c | The main diagnoses, therapeutic interventions, and outcomes | Abstract, paragraph 2 |
| | 3d | Conclusion—What is the main "take-away" lesson(s) from this case? | Abstract, paragraph 3 |
| Introduction | 4 | One or two paragraphs summarizing why this case is unique (**may include references**) | Background, paragraph 1 |
| Patient Information | 5a | De-identified patient specific information. | Case presentation, paragraph 1 |
| | 5b | Primary concerns and symptoms of the patient. | Case presentation, paragraph 1 |
| | 5c | Medical, family, and psycho-social history including relevant genetic information | N/A |
| | 5d | Relevant past interventions with outcomes | N/A |
| Clinical Findings | 6 | Describe significant physical examination (PE) and important clinical findings. | Case presentation, paragraph 1 |
| Timeline | 7 | Historical and current information from this episode of care organized as a timeline | N/A |
| Diagnostic Assessment | 8a | Diagnostic testing (such as PE, laboratory testing, imaging, surveys). | Case presentation, paragraph 1 |
| | 8b | Diagnostic challenges (such as access to testing, financial, or cultural) | N/A |
| | 8c | Diagnosis (including other diagnoses considered) | Case presentation, paragraph 1 |
| | 8d | Prognosis (such as staging in oncology) where applicable | Case presentation, paragraph 2 |
| Therapeutic Intervention | 9a | Types of therapeutic intervention (such as pharmacologic, surgical, preventive, self-care) | Case presentation, paragraph 2 |
| | 9b | Administration of therapeutic intervention (such as dosage, strength, duration) | Case presentation, paragraph 2 |
| | 9c | Changes in therapeutic intervention (with rationale) | N/A |
| Follow-up and Outcomes | 10a | Clinician and patient-assessed outcomes (if available) | Case presentation, paragraph 2 |
| | 10b | Important follow-up diagnostic and other test results | Case presentation, paragraph 2 |
| | 10c | Intervention adherence and tolerability (How was this assessed?) | N/A |
| | 10d | Adverse and unanticipated events | N/A |
| Discussion | 11a | A scientific discussion of the strengths AND limitations associated with this case report | Discussion, paragraph 1 |
| | 11b | Discussion of the relevant medical literature **with references**. | Discussion, paragraph 2 and 3 |
| | 11c | The scientific rationale for any conclusions (including assessment of possible causes) | Discussion, paragraph 4 |
| | 11d | The primary "take-away" lessons of this case report (without references) in a one paragraph conclusion | Discussion, paragraph 4 |
| Patient Perspective | 12 | The patient should share their perspective in one to two paragraphs on the treatment(s) they received | N/A |
| Informed Consent | 13 | Did the patient give informed consent? Please provide if requested | Yes ☑ No ☐ |
